# A Prospective Population Study of Resting Heart Rate and Peak Oxygen Uptake (the HUNT Study, Norway)

**DOI:** 10.1371/journal.pone.0045021

**Published:** 2012-09-18

**Authors:** Javaid Nauman, Stian Thoresen Aspenes, Tom Ivar Lund Nilsen, Lars J. Vatten, Ulrik Wisløff

**Affiliations:** 1 The K.G. Jebsen Center of Exercise in Medicine at Department of Circulation and Medical Imaging, Faculty of Medicine, Norwegian University of Science and Technology, Trondheim, Norway; 2 The Human Movement Science Programme, Faculty of Social Sciences and Technology Management, Norwegian University of Science and Technology, Trondheim, Norway; 3 Department of Public Health, Faculty of Medicine, Norwegian University of Science and Technology, Trondheim, Norway; Innsbruck Medical University, Austria

## Abstract

**Objectives:**

We assessed the prospective association of resting heart rate (RHR) at baseline with peak oxygen uptake (VO_2peak_) 23 years later, and evaluated whether physical activity (PA) could modify this association.

**Background:**

Both RHR and VO_2peak_ are strong and independent predictors of cardiovascular morbidity and mortality. However, the association of RHR with VO_2peak_ and modifying effect of PA have not been prospectively assessed in population studies.

**Methods:**

In 807 men and 810 women free from cardiovascular disease both at baseline (1984–86) and follow-up 23 years later, RHR was recorded at both occasions, and VO_2peak_ was measured by ergospirometry at follow-up. We used Generalized Linear Models to assess the association of baseline RHR with VO_2peak_, and to study combined effects of RHR and self-reported PA on later VO_2peak_.

**Results:**

There was an inverse association of RHR at baseline with VO_2peak_ (p<0.01). Men and women with baseline RHR greater than 80 bpm had 4.6 mL·kg^−1^·min^−1^ (95% confidence interval [CI], 2.8 to 6.3) and 1.4 mL·kg^−1^·min^−1^ (95% CI, −0.4 to 3.1) lower VO_2peak_ at follow-up compared with men and women with RHR below 60 bpm at baseline. We found a linear association of change in RHR with VO_2peak_ (p = 0.03), suggesting that a decrease in RHR over time is likely to be beneficial for cardiovascular fitness. Participants with low RHR and high PA at baseline had higher VO_2peak_ than inactive people with relatively high RHR. However, among participants with relatively high RHR and high PA at baseline, VO_2peak_ was similar to inactive people with relatively low RHR.

**Conclusion:**

RHR is an important predictor of VO_2peak_, and serial assessments of RHR may provide useful and inexpensive information on cardiovascular fitness. The results suggest that high levels of PA may compensate for the lower VO_2peak_ associated with a high RHR.

## Introduction

Resting heart rate (RHR) [1]–[3] and cardiorespiratory fitness [4]–[6] are strong and independent predictors of overall and cardiovascular morbidity and mortality. The results of epidemiological studies suggest that RHR is a low-tech and expedient method to estimate cardiorespiratory fitness, as indicated by peak oxygen uptake (VO_2peak_), and RHR is inversely associated with objectively measured VO_2peak_ [7]–[9].

No prospective study has assessed whether measured RHR at a certain baseline is associated with VO_2peak_ several years later. If high RHR is associated with lower VO_2peak_ later in life, then RHR may be useful in identifying people at increased risk of cardiovascular disease. Such an association may also advance our understanding of the physiological pathways that are involved in the regulation of cardiorespiratory fitness.

Cardiorespiratory fitness is a modifiable factor that is associated with physical activity [6], [10], and a high level of physical activity is associated with low RHR [11], [12]. Therefore, the association of RHR with cardiorespiratory fitness could be modified by physical activity level. To our knowledge, the combined effects of RHR and physical activity in relation to subsequent VO_2peak_ have not been previously studied.

Therefore, we have prospectively assessed the association of RHR measured at baseline in the mid 1980s with peak oxygen uptake measured 23 years later, and evaluated whether physical activity could modify the association of RHR with peak oxygen uptake.

## Methods

### Study Participants

The Nord-Trøndelag Health Study (the HUNT Study) is a large population based health survey in Norway where clinical measurements, medical and personal histories, exposure variables and biological materials have been collected in three consecutive waves from 1984 to 2008. Participants in the present study attended the first (HUNT 1, 1984–1986) and third wave (HUNT 3, 2006–2008), and attended a sub-study of HUNT 3 that included objective measurements of VO_2peak_. A detailed description of the participants in HUNT 3 Fitness project is described elsewhere [13]. In present study, we included 1617 participants (807 men and 810 women) who were all free from known heart or lung-disease at the time of HUNT 3 (i.e. had not had squeaky or heavy breathing for the past 12 months before the VO_2peak_-test, never had asthma, chronic bronchitis, COPD, sarcoidosis, heart disease, angina or cerebral infarction, and had never used antihypertensive medication), never had cancer, were not pregnant or blind, and did not have any other medical contraindication or orthopaedic limitation.

### Clinical Measurements

In addition to standardized measurements of RHR, the baseline examination also included measures of blood pressure, height and weight. Body mass index (BMI) was calculated as weight divided by height squared (kg•m^−2^). In HUNT 1, the RHR was measured by palpating the radial pulse over a period of 15 seconds with a stop-watch, after at least 4 minutes of seated rest. If the pulse was irregular or difficult to count, the test was extended to 30 seconds, if necessary with the aid of a stethoscope placed over the heart. In HUNT 3 Fitness project, RHR was recorded as the lowest heart rate by 3-point echocardiography (GE Healthcare, USA) during supine rest for 10 minutes in a dim lit, quiet room.

In HUNT 3, an individualized protocol [14] was applied to measure maximal oxygen uptake. Each test-subject was familiarized with treadmill walking during the warm-up of 8–10 minutes, also to ensure safety and avoid handrail grasp when this was not absolutely necessary. Oxygen uptake kinetics were measured directly by a portable mixing chamber gas-analyzer (MetaMax II, Cortex, Leipzig, Germany) with the participants wearing a tight face mask (Hans Rudolph, Germany) connected to the MetaMax II. When the participants reached an oxygen consumption that was stable over 30 seconds, inclination (1–2% each step) or velocity (0.5–1 km·h^−1^) on the treadmill was increased depending on the appearance of and feedback from the participants until exhaustion. A maximal test was achieved with a respiratory quotient of 1.05 or higher or when the oxygen uptake did not increase >2.0 mL· kg^−1^· min^−1^ despite increased workload or before the participant disembarked the treadmill. A total of 18% of the participants did not achieve maximal oxygen uptake, and therefore, the term VO_2peak_ was used, and measured as litres of oxygen uptake per minute (L·min^−1^), and subsequently calculated relative to body mass (mL·kg^−1^·min^−1^).

### Questionnaire-based Information

Information on physical activity was obtained from self-administered questionnaire applied in both HUNT 1 [15] and HUNT 3 [13]. The questionnaires included three questions: Question 1: “How frequently do you exercise?”, with the response options “Never” (0), “Less than once a week” (0), “Once a week” (1), “2–3 times per week” (2.5) and “Almost every day” (5). Question 2: “If you exercise as frequently as once or more times a week: How hard do you push yourself?” with the response options: “I take it easy without breaking a sweat or losing my breath” (1), “I push myself so hard that I lose my breath and break into sweat” (2) and “I push myself to near exhaustion” (3). Question 3: “How long does each session last?”, with the response options: “Less than 15 minutes” (0.1), “15–29 minutes” (0.38), “30 minutes to 1 hour” (0.75) and “More than 1 hour” (1.0). Each participant’s response to the questions about exercise frequency, intensity and duration (i.e. numbers in parentheses) were multiplied to calculate a physical activity index score (PAI). As the second (intensity of exercise) and third (duration of exercise) question only addressed people who exercised at least once a week, both “Never” and “Less than once a week” were considered inactive participants and yielded an index score of zero. Participants with a zero score were categorized as inactive, and the remaining participants were classified into three equally sized groups (tertiles) based on the sex-specific distribution of score values (i.e., low, medium or high PAI). The physical activity questions (frequency, intensity and duration) have been shown to provide a reproducible measure of leisure time physical activity, and PAI has been found highly reliable [13], [15].

Habitual smoking, alcohol consumption during last two weeks, and level of education were self-reported from the questionnaires in HUNT 1 & 3. Participants were divided into three categories depending on their smoking habits (never smoker, former smoker or daily smoker), alcohol consumption (0, 1–4 times, ≥5 times per two weeks), and into three categories depending on their level of education (9 year primary school or less, 10–12 year primary school, or university college).

### Ethics Statement

The study was approved by the Regional committee for medical research ethics, the Norwegian Data Inspectorate, and by the national Directorate of Health. The study is in conformity with Norwegian laws and the Helsinki declaration, and all participants signed a document of consent.

### Statistical Analyses

Descriptive data are presented as means and standard deviations (SD), and means were compared using paired samples t-tests for continuous variables, and by Wilcoxon signed-rank test for categorical variables. The RHR measurement both at HUNT 1 and HUNT 3 were classified into four categories (<60 bpm, 60–70 bpm, 71–80 bpm or >80 bpm). The choice of these cut-offs were made ‘a priori’ based on the similar cut-offs in other studies of RHR and cardiovascular risk [3], [16]. We used generalized linear model analyses [17], [18] to assess the association between RHR at HUNT 1 and objectively measured VO_2peak_ at HUNT 3. We also used RHR at HUNT 1 as a continuous variable and evaluated the association of each increment of 10 bpm with VO_2peak_. Furthermore, we studied if change in RHR from HUNT 1 to HUNT 3 was associated with VO_2peak_ at follow-up. To assess the association and to assess nonlinear trend, we used RHR as a continuous variable and following categories of change in RHR were used to allow the visual assessment of trend: (1) an increase of greater than 10 bpm; (2) an increase between 6 and 10 bpm; (3) a change from −5 to 5 bpm (reference); (4) a decrease between −6 to −10 bpm; and (5) a decrease more than −10 bpm.

In a separate analysis, we assessed the combined effect of RHR and physical activity at baseline on VO_2peak_ at follow-up. For this purpose, four categories of physical activity (inactive, low, medium or high), and four categories of heart rate (<60 bpm, 60–70 bpm, 71–80 or >80 bpm) were used. In the analysis, inactive participants with RHR >80 bpm served as the reference group.

All analyses were conducted for men and women separately, and we present both age adjusted and multivariable adjusted effect estimates with 95% confidence intervals. Multiple adjustments were made for age, smoking status, education, alcohol consumption, PAI, with adjustments for weight change from baseline to follow-up.

We performed additional analyses to assess the robustness of our findings. For example, we stratified RHR according to the baseline PAI, and also according to change in physical activity level over the time period. In other sensitivity analyses, we adjusted for RHR measured at HUNT 3. In analyses of change in RHR and VO_2peak_, we adjusted for change in physical activity from baseline to follow-up. All statistical tests were two-sided and *P*<0.05 was considered significant. The statistical analyses were conducted using Stata for Windows (Version 12.0 StataCorp LP).

## Results

Table 1 shows characteristics of the study participants at baseline (HUNT 1) and at follow-up (HUNT 3). During 23 years of follow-up, there was a mean reduction in RHR of 11.8 bpm (95% CI, 11.0 to 12.6) among men and 12.6 bpm (95% CI, 11.8 to 13.4) among women. The prevalence of smoking and the proportion of inactive participants were lower in HUNT 3 than at baseline in HUNT 1. Body weight has increased by 7.4 kg (95% CI, 6.9 to 7.9) and 8.3 kg (95% CI, 7.8 to 8.8) in men and women, respectively, and the corresponding increase in BMI was 2.6 kg·m^−2^ (95% CI, 2.4 to 2.7) and 3.3 kg·m^−2^ (95% CI, 3.1 to 3.5).

**Table 1:** Descriptive Statistics for Men and Women: Nord-Trøndelag Health Study (HUNT)^a^.

	Men (n = 807)				Women (n = 810)			
	HUNT 1		HUNT 3		HUNT 1		HUNT 3	
Age, years (SD)	35.3	(8.6)	58.1	(8.6)	35.0	(8.4)	57.8	(8.4)
Resting HR^b^, bpm (SD)	69.6	(11.4)	57.8	(8.9)	73.9	(11.1)	61.3	(9.6)
Weight^b^, kg (SD)	77.6	(8.5)	85.1	(10.6)	62.3	(8.4)	70.6	(10.9)
Height, cm (SD)	179.4	(5.8)	178.4	(6.0)	165.8	(5.3)	164.9	(5.6)
BMI^b^, kg·m^−2^ (SD)	24.1	(2.3)	26.7	(2.9)	22.6	(2.8)	25.9	(3.8)
VO_2peak_, mL·kg^−1^·min^−1^ (SD)	–		41.2	(8.3)	–		32.7	(6.5)
Systolic blood pressure^b^, mmHg (SD)	129.7	(11.7)	133.9	(14.5)	119.1	(11.2)	128.9	(16.6)
PAI b, No. (%)								
Inactive	260	(32.2)	115	(14.3)	252	(31.1)	75	(9.3)
Low	198	(24.5)	217	(26.9)	336	(41.5)	231	(28.5)
Medium	211	(26.2)	240	(29.7)	173	(21.4)	302	(37.3)
High	138	(17.1)	235	(29.1)	49	(6.0)	202	(24.9)
Smoking status^b^, No. (%)								
Current	171	(21.2)	127	(15.7)	230	(28.4)	169	(20.9)
Former	262	(32.5)	317	(39.3)	200	(24.7)	282	(34.8)
Never	374	(46.3)	354	(43.9)	380	(46.9)	353	(43.6)
Alcohol status^b^, No. (%)								
0	245	(30.3)	86	(10.7)	416	(51.3)	144	(17.8)
1–4	490	(60.7)	430	(53.3)	378	(46.7)	462	(56.9)
≥5	72	(9.0)	267	(33.0)	16	(2.0)	170	(21.0)

Abbreviations: HR, heart rate; bpm, beats per minute; BMI, body mass index (calculated as weight in kilograms divided by height in meters squared); VO_2peak_, peak oxygen uptake during a treadmill test; PAI, physical activity index.

a Data are expressed as mean (standard deviation), unless otherwise indicated.

b *P*<0.001.

### Baseline RHR and Future VO_2peak_

**Figure 1:**
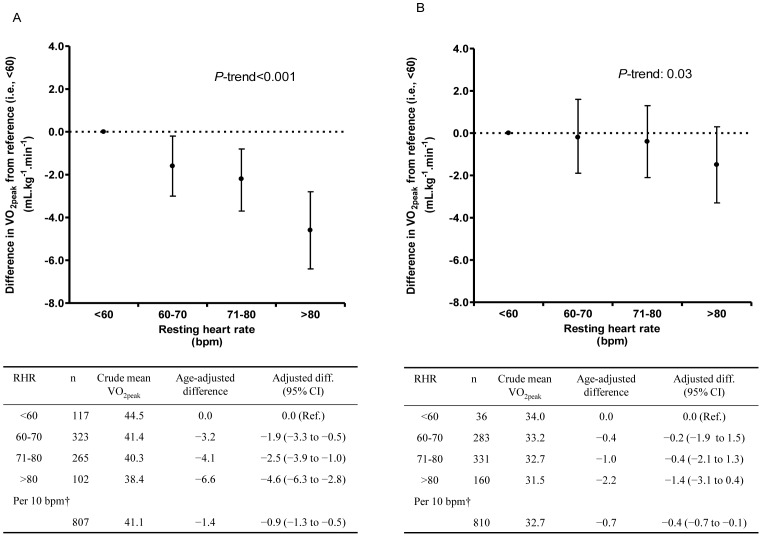
Difference in VO_2peak_ (measured at follow-up, 23 years later) across categories of resting heart rate (measured at baseline) with <60 beats per minute as reference (A: Men, B: Women). Adjusted for age, weight change, physical activity index (inactive, low, medium and high), smoking status (never, current and former), education (<10, 10–12 and ≥13 years) and alcohol status-frequency last two weeks (0, 1–4, and ≥5 times). The circles represent adjusted difference, and bars represent 95% confidence intervals. VO_2peak_, peak oxygen uptake; RHR, resting heart rate; bpm, beats per minute.

The association of RHR at baseline (HUNT 1) with VO_2peak_ at follow-up (HUNT 3) is shown in Figure 1. There was an inverse association of RHR with VO_2peak_, representing a weak yet significant correlation (r, −0.11 for women and −0.20 for men). Compared with men in the reference group (<60 bpm in HUNT 1), a heart rate higher than 80 bpm was associated with lower VO_2peak_ (4.6 mL·kg^−1^·min^−1^, 95% CI, 2.8 to 6.3) at follow-up (Figure 1, panel A). In women, a heart rate of >80 bpm at baseline was associated with lower VO_2peak_ (1.4 mL·kg^−1^·min^−1^, 95% CI, −0.4 to 3.1) at follow-up, compared with a heart rate lower than 60 bpm at baseline (Figure 1, panel B). For each increment of 10 bpm in RHR, the adjusted difference in VO_2peak_ was lower both in men (0.9 mL·kg^−1^·min^−1^, 95% CI, 0.5 to 1.3) and women (0.4 mL·kg^−1^·min^−1^, 95% CI, 0.1 to 0.7). In an additional analysis of RHR stratified according to the baseline PAI, and also according to change in physical activity level over the time period, RHR at baseline was associated with VO_2peak_, independent of physical activity status both at baseline and follow-up (Table S1). We also adjusted for the RHR at follow-up to assess the association of baseline RHR with VO_2peak_, and found that results were very similar to the results obtained without this adjustment (Figure S1).

### Temporal Changes in RHR and VO_2peak_

**Figure 2:**
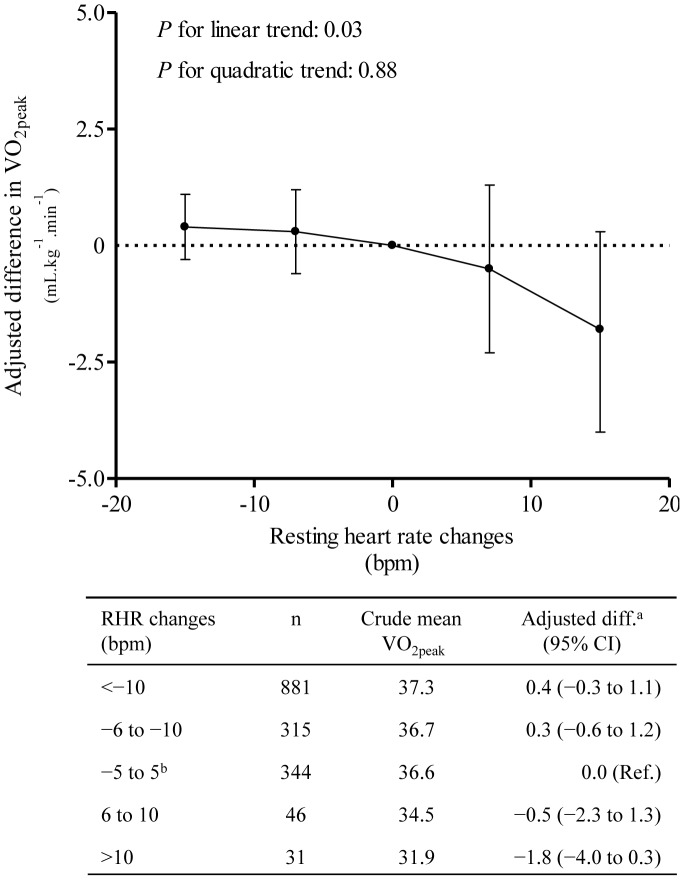
Difference in VO_2peak_ by changes in resting heart rate. ^a^Adjusted for age, sex, weight change, physical activity index (inactive, low, medium and high), smoking status (never, current and former), education (<10, 10–12 and ≥13 years) and alcohol status-frequency last two weeks (0, 1–4, and ≥5 times). ^b^Reference group The circles represent adjusted difference, and bars represent 95% confidence intervals. VO_2peak_, peak oxygen uptake; RHR, resting heart rate; bpm, beats per minute.

We also assessed whether changes in RHR from baseline to follow-up are associated with VO_2peak_ (Table 2). We found that an increase in RHR from baseline to follow-up was associated with lower VO_2peak_ at follow-up. Compared with men with RHR lower than 60 bpm at both occasions, the VO_2peak_ was 6.3 mL·kg^−1^·min^−1^ (95% CI, 1.0 to 11.7) lower for men with RHR between 71 and 80 bpm at baseline but higher than 80 bpm at follow-up. The corresponding decrease in VO_2peak_ among women was 1.3 mL·kg^−1^·min^−1^ (95% CI, −2.2 to 4.8). In the analyses using RHR as continuous variable, we found a linear association between change in RHR and VO_2peak_ (Figure 2; *P* for linear trend, 0.03 and *P* for quadratic trend, 0.88). The sensitivity analysis performed with additional controlling for change in physical activity from baseline to follow-up showed that the results materially did not change compared to results without this adjustment (Table S2 & S3).

**Table 2:** Adjusted differences in VO_2peak_ (mL·kg^−1^·min^−1^) from HUNT 3 according to resting heart rate in HUNT 1 and HUNT 3.

	Resting heart rate, HUNT 3 (bpm)			
	<60	60–70	71–80	>80
Resting heart rate, HUNT 1 (bpm)				
Men				
<60				
n	112	4	1	–
mean (mL·kg^−1^·min^−1^)	44.9	36.5	33.6	–
Age adjusted diff.	0.0	−7.5	−8.4	–
^a^Adjusted diff.	0.0	−4.6	−2.8	–
(95% CI)	(Ref.)	(−11.0 to 1.8)	(−15.5 to 9.9)	–
60–70				
n	234	74	12	3
mean (mL·kg^−1^·min^−1^)	42.3	39.5	37.4	40.3
Age adjusted diff.	−2.7	−5.1	−7.1	−6.4
^a^Adjusted diff.	−1.5	−3.5	−4.8	−4.4
(95% CI)	(−3.0 to −0.1)	(−5.4 to −1.6)	(−8.6 to −1.0)	(−11.7 to 3.0)
71–80				
n	135	110	14	6
mean (mL·kg^−1^·min^−1^)	41.2	39.9	38.6	32.7
Age adjusted diff.	−3.8	−4.8	−5.5	−10.9
^a^Adjusted diff.	−2.1	−3.0	−3.8	−6.3
(95% CI)	(−3.8 to −0.5)	(−4.7 to −1.2)	(−7.4 to −0.2)	(−11.7 to −1.0)
>80				
n	36	38	23	5
mean (mL·kg^−1^·min^−1^)	41.7	37.2	35.4	36.9
Age adjusted diff.	−4.8	−7.9	−8.1	−9.5
^a^Adjusted diff.	−2.9	−5.1	−7.2	−4.4
(95% CI)	(−5.3 to −0.4)	(−7.5 to −2.6)	(−10.1 to −4.2)	(−10.2 to 1.4)
Women				
<60				
n	24	10	2	–
mean (mL·kg^−1^·min^−1^)	35.5	29.4	38.2	–
Age adjusted diff.	0.0	−4.7	3.5	–
^a^Adjusted diff.	0.0	−3.6	1.1	–
(95% CI)	(Ref.)	(−7.3 to −0.0)	(−6.0 to 8.1)	–
60–70				
n	162	100	17	4
mean (mL·kg^−1^·min^−1^)	34.3	31.9	31.8	31.4
Age adjusted diff.	−0.8	−2.5	−1.7	−2.8
^a^Adjusted diff.	−0.7	−1.6	−2.3	−3.8
(95% CI)	(−2.8 to 1.4)	(−3.8 to 0.5)	(−5.4 to 0.7)	(−9.0 to 1.3)
71–80				
n	139	139	42	11
mean (mL·kg^−1^·min^−1^)	33.9	32.1	30.2	32.5
Age adjusted diff.	−1.4	−2.3	−3.9	−2.3
^a^Adjusted diff.	−0.7	−1.7	−2.1	−1.3
(95% CI)	(−2.8 to 1.4)	(−3.8 to 0.4)	(−4.6 to 0.3)	(−4.8 to 2.2)
>80				
n	38	78	29	15
mean (mL·kg^−1^·min^−1^)	34.4	30.9	30.0	31.1
Age adjusted diff.	−1.4	−3.6	−4.3	−4.8
^a^Adjusted diff.	−1.0	−3.0	−2.0	−2.6
(95% CI)	(−3.5 to 1.5)	(−5.3 to −0.8)	(−4.6 to 0.7)	(−5.7 to 0.6)

Abbreviations: VO_2peak_, peak oxygen uptake; bpm, beats per minute; CI, confidence interval.

Adjusted for age, weight change, smoking status (never, former, current), physical activity index (inactive, low, medium, high) education (<10, 10–12, >12 years), alcohol-frequency last two weeks (0, 1–4, ≥5 times).

### Modifying Effect of PA

The combined analysis of RHR and physical activity at baseline and VO_2peak_ at follow-up is presented in Table 3. The results showed that at every level of RHR, participants with a high level of physical activity at baseline had a high VO_2peak_ compared to inactive participants. The results also showed that men with high physical activity and a RHR lower than 60 bpm at baseline had higher VO_2peak_ (9.4 mL·kg^−1^·min^−1^, 95% CI, 6.7 to 12.2) at follow-up than men with no physical activity and RHR higher than 80 bpm. Among women with RHR higher than 80 bpm and who reported high physical activity, adjusted difference in VO_2peak_ was similar (1.2 mL·kg^−1^·min^−1^; 95% CI, −2.4 to 4.8) to inactive women with RHR lower than 60 bpm.

**Table 3:** Adjusted differences in VO_2peak_ (mL·kg^−1^·min^−1^) from HUNT 3 in combined categories of resting heart rate and physical activity (PA) from HUNT 1.

	Physical activity index, HUNT 1			
	Inactive	Low	Medium	High
Resting heart rate, HUNT 1 (bpm)				
Men				
>80				
n	49	25	14	14
mean (mL·kg^−1^·min^−1^)	36.5	39.4	38.5	42.9
Age adjusted diff.	0.0	3.6	1.2	6.3
aAdjusted diff.	0.0	2.4	0.3	5.6
(95% CI)	Ref.	(−0.7 to 5.6)	(−3.5 to 4.2)	(1.8 to 9.5)
71–80				
n	105	76	57	27
mean (mL·kg^−1^·min^−1^)	38.2	41.3	41.8	42.7
Age adjusted diff.	2.2	5.3	6.3	6.4
aAdjusted diff.	2.0	4.0	5.3	5.7
(95% CI)	(−0.2 to 4.2)	(1.6 to 6.4)	(2.8 to 7.8)	(2.7 to 8.8)
60–70				
n	93	81	94	55
mean (mL·kg^−1^·min^−1^)	39.5	41.0	41.9	44.4
Age adjusted diff.	2.7	5.3	6.8	7.4
aAdjusted diff.	2.7	4.7	5.6	5.7
(95% CI)	(0.5 to 4.9)	(2.4 to 7.0)	(3.3 to 7.9)	(3.2 to 8.3)
<60				
n	13	16	46	42
mean (mL·kg^−1^·min^−1^)	41.6	41.7	42.5	48.7
Age adjusted diff.	5.6	6.1	7.8	11.2
aAdjusted diff.	5.4	6.3	6.2	9.4
(95% CI)	(1.4 to 9.3)	(2.7 to 10.0)	(3.6 to 8.9)	(6.7 to 12.2)
Women				
>80				
n	54	67	31	8
mean (mL·kg^−1^·min^−1^)	30.6	31.8	32.9	31.0
Age adjusted diff.	0.0	1.1	2.4	1.5
aAdjusted diff.	0.0	0.1	1.2	1.2
(95% CI)	Ref.	(−1.7 to 1.8)	(−0.9 to 3.4)	(−2.4 to 4.9)
71–80				
n	109	143	62	17
mean (mL·kg^−1^·min^−1^)	32.4	31.7	34.5	35.2
Age adjusted diff.	1.4	1.7	4.1	4.4
aAdjusted diff.	0.9	0.9	2.8	2.4
(95% CI)	(−0.7 to 2.5)	(−0.7 to 2.4)	(1.0 to 4.6)	(−0.3 to 5.1)
60–70				
n	81	112	70	20
mean (mL·kg^−1^·min^−1^)	32.0	32.2	34.3	40.3
Age adjusted diff.	1.3	2.2	4.1	8.4
aAdjusted diff.	0.7	1.0	2.4	6.0
(95% CI)	(−1.0 to 2.3)	(−0.6 to 2.6)	(0.7 to 4.2)	(3.5 to 8.5)
<60				
n	8	14	10	4
mean (mL·kg^−1^·min^−1^)	32.9	32.3	34.2	41.3
Age adjusted diff.	2.7	1.8	4.1	6.8
aAdjusted diff.	1.2	1.4	3.2	3.0
(95% CI)	(−2.4 to 4.9)	(−1.5 to 4.3)	(−0.1 to 6.5)	(−2.0 to 8.0)

Abbreviations: VO_2peak_, peak oxygen uptake; bpm, beats per minute.

a Adjusted for age, weight change, smoking status (never, former, current), education (<10, 10–12, >12 years), alcohol-frequency last two weeks (0, 1–4, ≥5 times).

## Discussion

In this prospective study of men and women, we found that low RHR at baseline was a strong predictor of high VO_2peak_ 23 years later. We also found that an increase in RHR from baseline to follow-up was associated with lower VO_2peak_, and that high physical activity at baseline was associated with higher VO_2peak_ levels at follow-up, irrespective of high RHR at baseline.

Our findings are consistent with the results of cross sectional studies that have suggested an inverse association of RHR with VO_2peak_ [7]–[9]. We observed on average 5.5 mL·kg^−1^·min^−1^ (corresponding to ≈ 1.5 metabolic equivalent, MET) lower VO_2peak_ at follow-up in men and women who had high RHR (>80 bpm) compared to low RHR (<60 bpm) at baseline. In other prospective studies, it has been suggested that a decrease of 1 MET (3.5 mL·kg^−1^·min^−1^) may be associated with increased risk of diabetes, hypertension and the metabolic syndrome [19], [20], whereas a corresponding increase has been associated with a lower risk of developing metabolic syndrome [20], and lower risk of all-cause [4] and cardiovascular death [5]. Since VO_2peak_ is a strong predictor of cardiovascular health [4], [20], [21], our results therefore suggest that RHR measurements may be a useful marker for cardiovascular risk.

We found that change in RHR from baseline to follow-up was associated with VO_2peak_ at follow-up. Participants with RHRs between 71–80 bpm at baseline and higher than 80 bpm at follow-up had on average 4.0 ml·kg^−1^·min^−1^ (95% CI, 1.0 to 6.9) lower VO_2peak_, compared to those with low RHR at both occasions. We observed a linear association between longitudinal changes in RHR and VO_2peak_ (*P*-trend, 0.03). These findings suggest that a decrease in RHR over time is likely to be beneficial in relation to cardiovascular fitness. Other studies have also shown a favourable effect of heart rate reduction with overall cardiovascular health in the general population and in patients with cardiovascular disease [22], [23]. Moreover, the change in RHR is associated with physical activity status, and people with increased level of physical activity during a time period have low RHR [11], as also observed in our study (Table S4). However, we found that the association between change in RHR and VO_2peak_ was not substantially altered after additional controlling of change in physical activity during the follow-up. Other sensitivity analyses showed that an increase in physical activity from baseline to follow-up was associated with higher VO_2peak_ only in those participants with a decrease in RHR over the time period (Table S5). These results suggest that longitudinal assessment of RHR may provide useful and inexpensive information on cardiovascular fitness, independent of other risk factors.

It has been suggested that variations in RHR and VO_2peak_ may be dependent on sympathovagal balance where higher cardiorespiratory fitness and lower RHR are associated with enhanced vagal activity [24]–[27]. Thus, parasympathetic induced modulation could be a plausible explanation for the inverse association of RHR and VO_2peak_.

We found a combined effect of RHR and physical activity on VO_2peak_, and observed that participants with low RHR and high physical activity had higher VO_2peak_ compared with inactive people with high RHR. We also found that participants with relatively high RHR and high physical activity at baseline had a VO_2peak_ that was similar to people with relatively low RHR and no physical activity. These findings suggest that people with high RHR may increase their VO_2peak_ by engaging in physical activity. Previous studies have shown a favourable effect of physical activity on the association of heart rate and cardiovascular risk [28], [29], and data suggest that high levels of baseline physical activity among women may attenuate the risk of death form coronary heart disease associated with high baseline RHR [28]. These effects of physical activity on cardiovascular health and fitness may correspond to a large number of physiological changes including effects on heart – increased parasympathetic activity and decreased sympathetic activity, coupled with a possible reduction in intrinsic heart rate [10], [12], but also exercise-induced adaptations in skeletal muscle, systemic circulation and lung capacity [10], [30], [31]. Genes may also contribute to the observed level of VO_2peak_ associated with RHR [32]. Nonetheless, further research is warranted to better understand the modifying effect of physical activity on the association of RHR with VO_2peak_.

The relatively large (n = 1,617) population, the prospective nature of the study, and the long follow-up (23 years) are important features that strengthen the validity of our results. At follow-up, we used directly measured VO_2peak_ based on an individual protocol, which is considered the most accurate method to define cardiorespiratory fitness. It is possible that changes in other risk factors from baseline to follow-up could have influenced the association of RHR with VO_2peak_, but it is reassuring that the results were not substantially altered after adjustments for changes in many relevant risk factors that we had information about.

### Study Limitations

Our study was restricted to people who were apparently free from any heart or lung disease both at baseline and at follow-up, and had never used any antihypertensive medications. This should have covered people with atrial fibrillation or any other arrhythmias. However, important lifestyle factors, including the prevalence of smoking, educational level and physical activity level in this study population may differ from those of the general population. In relation to generalizability, this may be a limitation of our results. Baseline VO_2peak_ assessment was not conducted in present study and it would have strengthened the results if such information had been available. In HUNT 1, RHR was assessed by palpating the radial pulse over a period of 15 seconds, and therefore is likely to be prone to measurement error. However, the test was extended to 30 seconds and if necessary a stethoscope was placed over heart when pulse was difficult to count. In HUNT 3, RHR was assessed by echocardiography recordings, and one can expect lower heart rates using this procedure; however, previous studies [33], [34] have shown that RHR assessment by palpation or by auscultation have strong correlation with electrocardiographic measurements, and could be an adequate surrogate measure in the absence of electrocardiography. The temporal decrease in RHR in present study is consistent with other longitudinal studies [11], [35] that have shown a considerable decline in RHR over the years, independent of age and presence of other cardiovascular risk factors. In Paris Prospective study [35], mean RHR decreased by 5 bpm in men and 7 bpm in women, over 16 years. A curvilinear association was reported between RHR and year of entry to university among first year undergraduates from 1949–2004, where RHR initially decreased (9.5 bpm in men, and 10.4 bpm in women) and then increased from the mid-1980s until the end of follow-up [11]. Moreover, the difference in methods to measure the RHR at baseline and follow-up may lead to non-differential bias and most likely to yield underestimates of the true effects rather than a spurious overestimates. Other unmeasured or unknown factors, such as cholesterol, may have confounded the association of RHR with VO_2peak_. However, a weak association of total cholesterol with VO_2peak_ has been reported [13].

### Clinical Implications

Heart rate is used in the assessment of cardiovascular risk [36], cardiorespiratory fitness [7], [8], and has also been included in clinical scores to predict mortality from all causes [37]. Our findings extend the evidence that RHR is an important predictor for cardiovascular fitness, and our results support the hypothesis that high RHR is associated with increased cardiovascular risk in an apparently healthy population.

## Supporting Information

Figure S1 **Adjusted differences^†^ in VO_2peak_ (mL·kg^−1^·min^−1^) across categories of resting heart rate after additional adjustment of resting heart rate at HUNT 3.** Adjusted for age, sex, weight change, resting heart rate at HUNT 3, physical activity index (inactive, low, medium and high), smoking status (never, current and former), education (<10, 10–12 and ≥13 years) and alcohol status-frequency last two weeks (0, 1–4, and ≥5 times). The circles represent adjusted difference, and bars represent 95% confidence intervals. VO_2peak_, peak oxygen uptake; RHR, resting heart rate; bpm, beats per minute. ^†^To increase the statistical power of analyses, men and women were pooled together, adjusting for sex.
(TIF)

Table S1 **Adjusted differences^†^ in VO_2peak_ (mL·kg^−1^·min^−1^) according to resting heart rate and physical activity.** Abbreviations: VO_2peak_, peak oxygen uptake; bpm, beats per minute. Adjusted for age, sex, weight change, smoking status (never, former, current), education (<10, 10–12, >12 years), alcohol-frequency last two weeks (0, 1–4, ≥5 times). ^†^To increase the statistical power of analyses, men and women were pooled together, adjusting for sex.
(DOC)

Table S2 **Adjusted difference in VO_2peak_ (mL·kg^−1^·min^−1^) by change in resting heart rate, after additional controlling for change in physical activity.** Abbreviations: VO_2peak_, peak oxygen uptake; bpm, beats per minute; CI, confidence interval. Adjusted for age, physical activity index (inactive, low, medium, high, weight change, smoking status (never, former, current), education (<10, 10–12, >12 years), alcohol-frequency last two weeks (0, 1–4, ≥5 times), and physical activity changes from baseline to follow-up (unchanged, decreased, increased).
(DOC)

Table S3 **Changes in resting heart rate and VO_2peak_ after additional controlling of change in physical activity.** Abbreviations: VO_2peak_, peak oxygen uptake; bpm, beats per minute; CI, confidence interval. ^a^Adjusted for age, sex, weight change, smoking status (never, former, current), physical activity index (inactive, low, medium, high) education (<10, 10–12, >12 years), alcohol-frequency last two weeks (0, 1–4, ≥5 times), changes in physical activity from baseline to follow-up (unchanged, decreased, increased).
(DOC)

Table S4 **Change in physical activity and change in resting heart rate.** Abbreviations: PAI, physical activity index; RHR, resting heart rate; CI, confidence interval.
(DOC)

Table S5 **Adjusted difference in VO_2peak_ according to change in physical activity and change in resting heart rate.** Adjusted for age, sex, weight change, smoking status (never, former, current), education (<10, 10–12, >12 years), alcohol-frequency last two weeks (0, 1–4, ≥5 times).
(DOC)
