# Supplementary figures and images for: A Prospective Population Study of Resting Heart Rate and Peak Oxygen Uptake (the HUNT Study, Norway)
Source: PLoS One. 2012 Sep 18;7(9):e45021. doi: 10.1371/journal.pone.0045021 (PMC3445602; doi:10.1371/journal.pone.0045021)

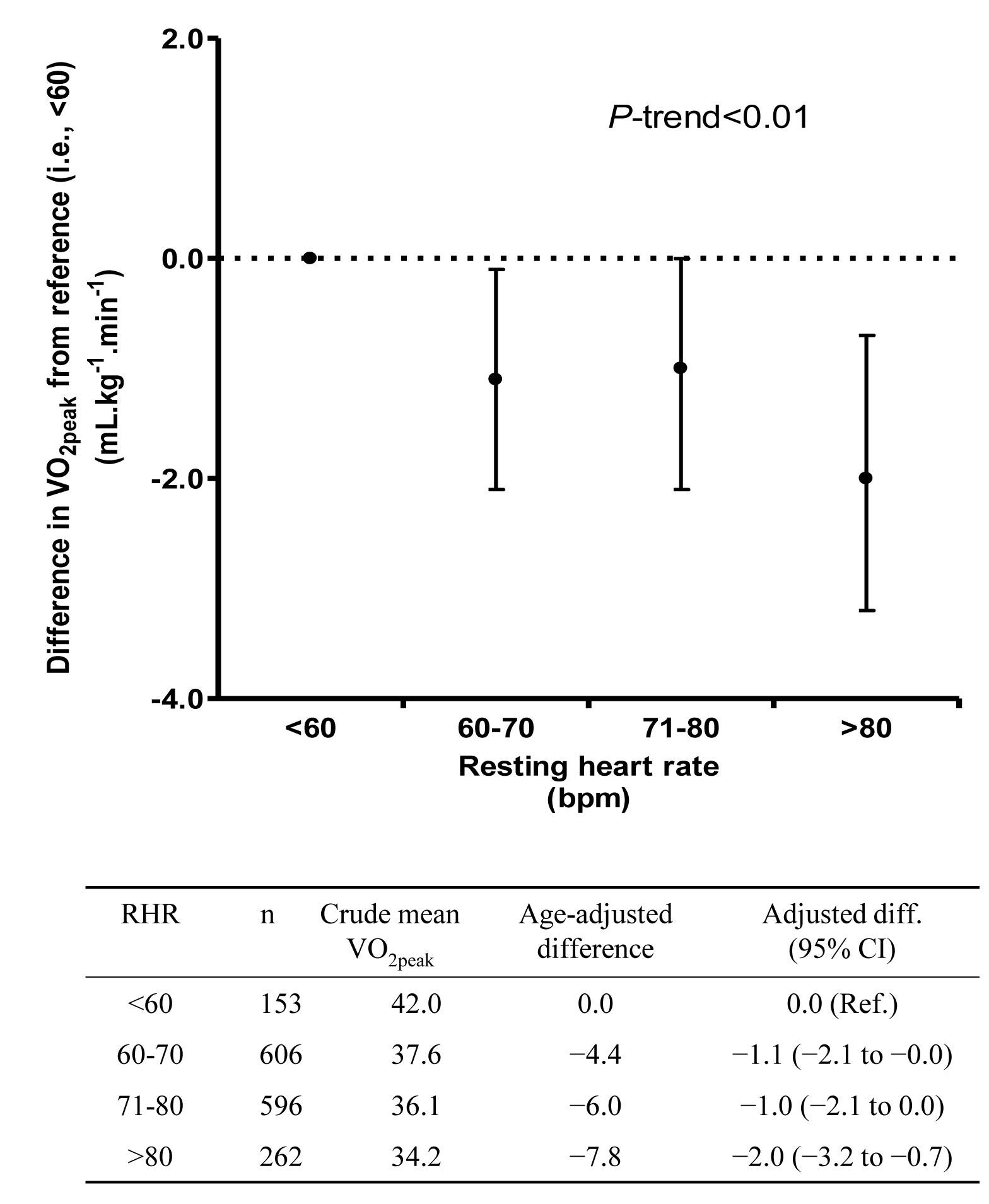

Supplement: Figure S1 — Adjusted differences† in VO2peak (mL·kg−1·min−1) across categories of resting heart rate after additional adjustment of resting heart rate at HUNT 3. Adjusted for age, sex, weight change, resting heart rate at HUNT 3, physical activity index (inactive, low, medium and high), smoking status (never, current and former), education (<10, 10–12 and ≥13 years) and alcohol status-frequency last two weeks (0, 1–4, and ≥5 times). The circles represent adjusted difference, and bars represent 95% confidence intervals. VO2peak, peak oxygen uptake; RHR, resting heart rate; bpm, beats per minute. †To increase the statistical power of analyses, men and women were pooled together, adjusting for sex. (TIF) [file pone.0045021.s001.tif]
