# Supplementary material for: A Prospective Population Study of Resting Heart Rate and Peak Oxygen Uptake (the HUNT Study, Norway)
Source: PLoS One. 2012 Sep 18;7(9):e45021. doi: 10.1371/journal.pone.0045021 (PMC3445602; doi:10.1371/journal.pone.0045021)
Supplement: Table S1 — Adjusted differences† in VO2peak (mL·kg−1·min−1) according to resting heart rate and physical activity. Abbreviations: VO2peak, peak oxygen uptake; bpm, beats per minute. Adjusted for age, sex, weight change, smoking status (never, former, current), education (<10, 10–12, >12 years), alcohol-frequency last two weeks (0, 1–4, ≥5 times). †To increase the statistical power of analyses, men and women were pooled together, adjusting for sex. (DOC) [file pone.0045021.s002.doc]

| **Table S1** Adjusted differences^†^ in VO_2peak_ (mL·kg^-1^·min^-1^) from HUNT 3 according to resting heart rate and physical activity | | | | | | |
| --- | --- | --- | --- | --- | --- | --- |
|  | | | Resting heart rate, HUNT 1 (bpm) | | | |
|  | | | <60 | 60-70 | 71-80 | >80 |
| Physical activity index, HUNT 1 | | | |  |  |  |
|  | Inactive | |  |  |  |  |
|  |  | n | 21 | 174 | 214 | 103 |
|  |  | Adjusted diff. | 0.0 | −2.5 | −3.0 | −4.6 |
|  |  | (95% CI) | (Ref.) | (−5.3 to 0.3) | (−5.8 to −0.2) | (−7.6 to −1.7) |
|  | Low | |  |  |  |  |
|  |  | n | 30 | 193 | 219 | 92 |
|  |  | Adjusted diff. | 0.0 | −2.0 | −2.8 | −4.4 |
|  |  | (95% CI) | (Ref.) | (−4.5 to 0.6) | (−5.3 to −0.2) | (−7.2 to −1.7) |
|  | Medium | |  |  |  |  |
|  |  | n | 56 | 164 | 119 | 45 |
|  |  | Adjusted diff. | 0.0 | −2.4 | −2.8 | −6.4 |
|  |  | (95% CI) | (Ref.) | (−4.5 to −0.3) | (−5.0 to −0.6) | (−9.1 to −3.6) |
|  | High | |  |  |  |  |
|  |  | n | 46 | 75 | 44 | 22 |
|  |  | Adjusted diff. | 0.0 | −4.3 | −6.0 | −5.6 |
|  |  | (95% CI) | (Ref.) | (−7.4 to −1.2) | (−9.6 to −2.5) | (−10.0 to −1.2) |
| Change in physical activity | | |  |  |  |  |
|  | Decreased | |  |  |  |  |
|  |  | n | 34 | 88 | 80 | 32 |
|  |  | Adjusted diff. | 0.0 | −3.5 | −3.3 | −7.0 |
|  |  | (95% CI) | (Ref.) | (−6.1 to −0.9) | (−6.0 to −0.6) | (−10.2 to −3.8) |
|  | Unchanged | |  |  |  |  |
|  |  | n | 63 | 210 | 185 | 81 |
|  |  | Adjusted diff. | 0.0 | −4.9 | −6.5 | −8.0 |
|  |  | (95% CI) | (Ref.) | (−7.0 to −2.8) | (−8.7 to −4.4) | (−10.5 to −5.5) |
|  | Increased | |  |  |  |  |
|  |  | n | 56 | 308 | 331 | 149 |
|  |  | Adjusted diff. | 0.0 | −3.0 | −4.0 | −5.5 |
|  |  | (95% CI) | (Ref.) | (−4.9 to −1.0) | (−5.9 to −2.1) | (−7.6 to −3.5) |
| Abbreviations: VO_2peak_, peak oxygen uptake; bpm, beats per minute | | | | | | |
| Adjusted for age, sex, weight change, smoking status (never, former, current), education (<10, 10-12, >12 years), alcohol-frequency last two weeks (0, 1-4, ≥5 times). | | | | | | |
| ^†^To increase the statistical power of analyses, men and women were pooled together, adjusting for sex. | | | | | | |
