# Supplementary material for: A Prospective Population Study of Resting Heart Rate and Peak Oxygen Uptake (the HUNT Study, Norway)
Source: PLoS One. 2012 Sep 18;7(9):e45021. doi: 10.1371/journal.pone.0045021 (PMC3445602; doi:10.1371/journal.pone.0045021)
Supplement: Table S2 — Adjusted difference in VO2peak (mL·kg−1·min−1) by change in resting heart rate, after additional controlling for change in physical activity. Abbreviations: VO2peak, peak oxygen uptake; bpm, beats per minute; CI, confidence interval. Adjusted for age, physical activity index (inactive, low, medium, high, weight change, smoking status (never, former, current), education (<10, 10–12, >12 years), alcohol-frequency last two weeks (0, 1–4, ≥5 times), and physical activity changes from baseline to follow-up (unchanged, decreased, increased). (DOC) [file pone.0045021.s003.doc]

| **Table S2** Adjusted difference in VO_2peak_ (mL·kg^-1^·min^-1^) by change in resting heart rate, after additional controlling for change in physical activity | | | | | | |
| --- | --- | --- | --- | --- | --- | --- |
|  | | | Resting heart rate, HUNT 3 (bpm) | | | |
|  | | | <60 | 60-70 | 71-80 | >80 |
| Resting heart rate, HUNT 1 (bpm) | | | |  |  |  |
| Men | | |  |  |  |  |
|  | <60 | |  |  |  |  |
|  |  | n | 112 | 4 | 1 | - |
|  |  | mean (mL·kg^-1^·min^-1^) | 44.9 | 36.5 | 33.6 | - |
|  |  | Age adjusted diff. | 0.0 | −7.5 | −8.4 | - |
|  |  | ^a^Adjusted diff. | 0.0 | −5.0 | −4.2 | - |
|  |  | (95% CI) | (Ref.) | (−11.1 to 1.0) | (−16.1 to 7.7) | - |
|  | 60-70 | |  |  |  |  |
|  |  | n | 234 | 74 | 12 | 3 |
|  |  | mean (mL·kg^-1^·min^-1^) | 42.3 | 39.5 | 37.4 | 40.3 |
|  |  | Age adjusted diff. | −2.7 | −5.1 | −7.1 | −6.4 |
|  |  | ^a^Adjusted diff. | −1.3 | −3.3 | −4.7 | −5.4 |
|  |  | (95% CI) | (−2.7 to 0.1) | (−5.1 to −1.5) | (−8.2 to −1.1) | (−12.3 to 1.5) |
|  | 71-80 | |  |  |  |  |
|  |  | n | 135 | 110 | 14 | 6 |
|  |  | mean (mL·kg^-1^·min^-1^) | 41.2 | 39.9 | 38.6 | 32.7 |
|  |  | Age adjusted diff. | −3.8 | −4.8 | −5.5 | −10.9 |
|  |  | ^a^Adjusted diff. | −1.7 | −2.4 | −3.3 | −6.0 |
|  |  | (95% CI) | (−3.3 to −0.2) | (−4.0 to −0.7) | (−6.7 to 0.0) | (−11.0 to −1.0) |
|  | >80 | |  |  |  |  |
|  |  | n | 36 | 38 | 23 | 5 |
|  |  | mean (mL·kg^-1^·min^-1^) | 41.7 | 37.2 | 35.4 | 36.9 |
|  |  | Age adjusted diff. | −4.8 | −7.9 | −8.1 | −9.5 |
|  |  | ^a^Adjusted diff. | −2.7 | −5.0 | −6.4 | −3.3 |
|  |  | (95% CI) | (−5.0 to −0.4) | (−7.3 to −2.7) | (−9.1 to −3.7) | (−8.8 to 2.1) |
| Women | | |  |  |  |  |
|  | <60 | |  |  |  |  |
|  |  | n | 24 | 10 | 2 | - |
|  |  | mean (mL·kg^-1^·min^-1^) | 35.5 | 29.4 | 38.2 | - |
|  |  | Age adjusted diff. | 0.0 | −4.7 | 3.5 | - |
|  |  | ^a^Adjusted diff. | 0.0 | −3.8 | −0.0 | - |
|  |  | (95% CI) | (Ref.) | (−7.3 to −0.2) | (−6.9 to 6.9) | - |
|  | 60-70 | |  |  |  |  |
|  |  | n | 162 | 100 | 17 | 4 |
|  |  | mean (mL·kg^-1^·min^-1^) | 34.3 | 31.9 | 31.8 | 31.4 |
|  |  | Age adjusted diff. | −0.8 | −2.5 | −1.7 | −2.8 |
|  |  | ^a^Adjusted diff. | −0.8 | −1.7 | −2.2 | −4.7 |
|  |  | (95% CI) | (−2.9 to 1.2) | (−3.8 to 0.4) | (−5.2 to 0.8) | (−9.8 to 0.3) |
|  | 71-80 | |  |  |  |  |
|  |  | n | 139 | 139 | 42 | 11 |
|  |  | mean (mL·kg^-1^·min^-1^) | 33.9 | 32.1 | 30.2 | 32.5 |
|  |  | Age adjusted diff. | −1.4 | −2.3 | −3.9 | −2.3 |
|  |  | ^a^Adjusted diff. | −0.7 | −1.9 | −2.0 | −0.9 |
|  |  | (95% CI) | (−2.8 to 1.4) | (−4.0 to 0.2) | (−4.4 to 0.4) | (−4.4 to 2.5) |
|  | >80 | |  |  |  |  |
|  |  | n | 38 | 78 | 29 | 15 |
|  |  | mean (mL·kg^-1^·min^-1^) | 34.4 | 30.9 | 30.0 | 31.1 |
|  |  | Age adjusted diff. | −1.4 | −3.6 | −4.3 | −4.8 |
|  |  | ^a^Adjusted diff. | −1.1 | −3.0 | −1.9 | −3.0 |
|  |  | (95% CI) | (−3.5 to 1.4) | (−5.2 to −0.8) | (−4.5 to 0.7) | (−6.1 to 0.1) |

Abbreviations: VO_2peak_, peak oxygen uptake; bpm, beats per minute; CI, confidence interval

Adjusted for age, physical activity index (inactive, low, medium, high, weight change, smoking status (never, former, current), education (<10, 10-12, >12 years), alcohol-frequency last two weeks (0, 1-4, ≥5 times), and physical activity changes from baseline to follow-up (unchanged, decreased, increased).
