# Supplementary material for: A Prospective Population Study of Resting Heart Rate and Peak Oxygen Uptake (the HUNT Study, Norway)
Source: PLoS One. 2012 Sep 18;7(9):e45021. doi: 10.1371/journal.pone.0045021 (PMC3445602; doi:10.1371/journal.pone.0045021)
Supplement: Table S3 — Changes in resting heart rate and VO2peak after additional controlling of change in physical activity. Abbreviations: VO2peak, peak oxygen uptake; bpm, beats per minute; CI, confidence interval. aAdjusted for age, sex, weight change, smoking status (never, former, current), physical activity index (inactive, low, medium, high) education (<10, 10–12, >12 years), alcohol-frequency last two weeks (0, 1–4, ≥5 times), changes in physical activity from baseline to follow-up (unchanged, decreased, increased). (DOC) [file pone.0045021.s004.doc]

| **Table S3** Changes in resting heart rate and VO_2peak_ after additional controlling of change in physical activity | | | | |
| --- | --- | --- | --- | --- |
| Change in RHR | n | Crude mean | ^a^Adjusted | (95% CI) |
| (bpm) |  | (mL·kg^-1^·min^-1^) | diff. |  |
| <−10 | 881 | 37.3 | 0.3 | (−0.4 to 1.0) |
| −6 to −10 | 314 | 36.7 | 0.2 | (−0.7 to 1.0) |
| −5 to 5 | 343 | 36.6 | 0.0 | (Ref.) |
| 6 to 10 | 46 | 34.5 | −0.8 | (−2.5 to 0.9) |
| >10 | 33 | 31.9 | −2.1 | (−4.2 to −0.1) |
| Abbreviations: VO_2peak_, peak oxygen uptake; bpm, beats per minute; CI, confidence interval | | | | |
| ^a^Adjusted for age, sex, weight change, smoking status (never, former, current), physical activity index (inactive, low, medium, high) education (<10, 10-12, >12 years), alcohol-frequency last two weeks (0, 1-4, ≥5 times), changes in physical activity from baseline to follow-up (unchanged, decreased, increased). | | | | |
