# Supplementary material for: A Prospective Population Study of Resting Heart Rate and Peak Oxygen Uptake (the HUNT Study, Norway)
Source: PLoS One. 2012 Sep 18;7(9):e45021. doi: 10.1371/journal.pone.0045021 (PMC3445602; doi:10.1371/journal.pone.0045021)
Supplement: Table S4 — Change in physical activity and change in resting heart rate. Abbreviations: PAI, physical activity index; RHR, resting heart rate; CI, confidence interval. (DOC) [file pone.0045021.s005.doc]

| Table S4 Change in physical activity and change in resting heart rate | | | |
| --- | --- | --- | --- |
|  |  | N | Change in RHR, bpm (95% CI) |
| HUNT 1, PAI | HUNT 3, PAI |  |  |
| Inactive | Inactive | 119 | −12.3 (−14.2 to −10.5) |
|  | Low | 172 | −14.2 (−15.9 to −12.5) |
|  | Medium | 134 | −13.3 (−15.3 to −11.2) |
|  | High | 87 | −16.6 (−19.4 to −13.7) |
| Low | Inactive | 40 | −13.7 (−16.2 to −11.2) |
|  | Low | 171 | −11.9 (−13.6 to −10.3) |
|  | Medium | 197 | −12.0 (−13.5 to −10.5) |
|  | High | 126 | −12.6 (−14.7 to −10.4) |
| Medium | Inactive | 20 | −15.4 (−21.4 to −9.4) |
|  | Low | 83 | −10.6 (−12.9 to −8.2) |
|  | Medium | 153 | −10.0 (−11.6 to −8.4) |
|  | High | 128 | −11.7 (−13.6 to −9.7) |
| High | Inactive | 11 | −8.3 (−13.8 to −2.7) |
|  | Low | 22 | −10.5 (−15.7 to −5.3) |
|  | Medium | 58 | −9.9 (−12.6 to −7.1) |
|  | High | 96 | −11.1 (−13.2 to −8.9) |

Abbreviations: PAI, physical activity index; RHR, resting heart rate; CI, confidence interval
