# Supplementary material for: A Prospective Population Study of Resting Heart Rate and Peak Oxygen Uptake (the HUNT Study, Norway)
Source: PLoS One. 2012 Sep 18;7(9):e45021. doi: 10.1371/journal.pone.0045021 (PMC3445602; doi:10.1371/journal.pone.0045021)
Supplement: Table S5 — Adjusted difference in VO2peak according to change in physical activity and change in resting heart rate. Adjusted for age, sex, weight change, smoking status (never, former, current), education (<10, 10–12, >12 years), alcohol-frequency last two weeks (0, 1–4, ≥5 times). (DOC) [file pone.0045021.s006.doc]

| Table S5 Adjusted difference in VO_2peak_ according to change in physical activity and change in resting heart rate | | | | | | | |
| --- | --- | --- | --- | --- | --- | --- | --- |
|  |  |  | Change in resting heart rate (bpm) | | | | |
|  |  | N | <−10 | −6 to −10 | −5 to 5 | 6 to 10 | >10 |
| HUNT 1, PAI | HUNT 3, PAI |  |  |  |  |  |  |
| Inactive | Inactive | 119 | n=66 | n=22 | *n=27* | n=2 | n=2 |
|  |  |  | 0.5 (−1.8 to 2.8) | 3.0 (0.1 to 5.9) | *0.0 (Ref.)* | 1.6 (−5.8 to 9.1) | −1.5 (−9.0 to 6.0) |
|  | Low | 172 | n=108 | n=27 | n=32 | n=4 | n=1 |
|  |  |  | 2.8 (0.6 to 5.0) | 1.9 (−0.8 to 4.7) | 1.9 (−0.8 to 4.5) | −0.2 (−5.7 to 5.3) | −4.9 (−15.5 to 5.6) |
|  | Medium | 134 | n=81 | n=22 | n=24 | n=3 | n=4 |
|  |  |  | 4.0 (1.7 to 6.3) | 5.1 (2.2 to 8.1) | 3.5 (0.7 to 6.4) | 1.2 (−5.0 to 7.5) | 5.5 (−0.0 to 11.0) |
|  | High | 87 | n=57 | n=15 | n=13 | n=1 | n=1 |
|  |  |  | 5.6 (3.3 to 8.0) | 3.7 (0.4 to 7.0) | 4.5 (1.1 to 8.0) | 1.7 (−8.7 to 12.1) | 5.6 (−4.8 to 15.9) |
|  |  |  |  |  |  |  |  |
| Low | Inactive | 40 | n= 27 | n=8 | n=5 | - | - |
|  |  |  | −0.0 (−2.6 to 2.6) | −2.1 (−6.1 to 1.9) | −1.7 (−6.6 to 3.2) |  |  |
|  | Low | 171 | n=86 | n=40 | *n=37* | n=4 | n=4 |
|  |  |  | 0.2 (−1.8 to 2.3) | 1.7 (−0.6 to 4.1) | *0.0 (Ref.)* | −0.1 (−5.6 to 5.4) | −0.2 (−5.6 to 5.2) |
|  | Medium | 197 | n=109 | n=41 | n=38 | n=5 | n=4 |
|  |  |  | 3.0 (1.0 to 5.0) | 1.7 (−0.6 to 4.0) | 1.8 (−0.6 to 4.2) | −1.5 (−6.4 to 3.4) | 0.2 (−5.2 to 5.6) |
|  | High | 126 | n=75 | n=17 | n=24 | n=7 | n=3 |
|  |  |  | 2.9 (0.8 to 5.0) | 3.2 (0.2 to 6.3) | 1.8 (−0.9 to 4.5) | 2.6 (−1.6 to 6.8) | −1.4 (−7.6 to 4.8) |
| Medium | Inactive | 20 | n=12 | n=5 | n=2 | n=1 | - |
|  |  |  | −4.3 (−8.0 to −0.7) | −5.2 (−10.5 to 0.0) | −3.1 (−11.1 to 4.8) | −4.1 (−15.2 to 6.9) |  |
|  | Low | 83 | n=40 | n=13 | n=25 | n=3 | n=2 |
|  |  |  | −5.1 (−7.6 to −2.6) | −5.4 (−8.9 to −1.8) | −4.8 (−7.7 to −2.0) | −0.4 (−7.0 to 6.2) | −7.1 (−15.1 to 0.8) |
|  | Medium | 153 | n=67 | n=40 | *n=37* | n=5 | n=4 |
|  |  |  | −1.3 (−3.5 to 0.9) | −1.2 (−3.7 to 1.3) | *0.0 (Ref.)* | −1.2 (−6.5 to 4.0) | −5.2 (−10.9 to 0.6) |
|  | High | 128 | n=65 | n=27 | n=28 | n=5 | n=3 |
|  |  |  | 1.0 (−1.3 to 3.2) | −0.1 (−2.9 to 2.7) | −0.9 (−3.6 to 1.9) | −1.3 (−6.5 to 3.9) | −0.8 (−7.3 to 5.8) |
| High | Inactive | 11 | n=3 | n=3 | n=5 | - | - |
|  |  |  | −12.8 (−21.7 to −3.8) | −13.4 (−22.1 to −4.7) | −7.6 (−14.6 to −0.6) |  |  |
|  | Low | 22 | n=9 | n=3 | n=10 | - | - |
|  |  |  | −5.6 (−11.8 to 0.7) | −15.6 (−24.2 to −7.1) | −10.1 (−15.4 to −4.8) |  |  |
|  | Medium | 58 | n=28 | n=11 | n=15 | n=3 | n=1 |
|  |  |  | −9.2 (−13.2 to −5.2) | −9.3 (−14.6 to −4.1) | −5.9 (−10.6 to −1.3) | −7.7 (−16.6 to 1.1) | −14.5 (−28.8 to −0.2) |
|  | High | 96 | n=48 | n=21 | *n=22* | n=3 | n=2 |
|  |  |  | −2.5 (−6.1 to 1.0) | −2.4 (−6.6 to 1.8) | *0.0 (Ref.)* | −2.8 (−11.4 to 5.7) | −5.4 (−15.7 to 4.9) |

Adjusted for age, sex, weight change, smoking status (never, former, current), education (<10, 10-12, >12 years), alcohol-frequency last two weeks (0, 1-4, ≥5 times)
